# Recent advances in understanding chronic myeloid leukemia: where do we stand?

**DOI:** 10.12703/r/10-35

**Published:** 2021-04-01

**Authors:** Rahul Kumar, Daniela S Krause

**Affiliations:** 1 Georg-Speyer-Haus, Institute for Tumor Biology and Experimental Therapy, 60596 Frankfurt, Germany; 2 German Cancer Research Center (DKFZ), Heidelberg, Germany; 3 German Cancer Consortium (DKTK), Heidelberg, Germany; 4 Frankfurt Cancer Institute, Frankfurt, Germany; 5 Faculty of Medicine, Johann Wolfgang Goethe University, Frankfurt, Germany

**Keywords:** Chronic myeloid leukemia, treatment-free remission, bone marrow microenvironment, immunological factors, autophagy, metabolism, epigenetics

## Abstract

While the need for complete eradication of leukemic stem cells (LSCs) in chronic myeloid leukemia may be controversial, it is agreed that remaining LSCs are the cause of relapse and disease progression. Current efforts are focused on the understanding of the persistence of immunophenotypically defined LSCs, which feature abnormalities in signaling pathways relating to autophagy, metabolism, epigenetics, and others and are influenced by leukemia cell-extrinsic factors such as the immune and bone marrow microenvironments. In sum, these elements modulate response and resistance to therapies and the clinical condition of treatment-free remission (TFR), the newly established goal in CML treatment, once the patient has achieved a durable molecular remission after treatment with tyrosine kinase inhibitors. Novel combination therapies based on these identified vulnerabilities of LSCs, aimed at the induction or maintenance of TFR, are being developed, while other research is directed at the elucidation of factors mediating progression to blast crisis.

## Introduction

Chronic myeloid leukemia (CML), a malignancy originating in hematopoietic stem cells (HSCs) in the chronic phase of the disease (Table 1) and characterized by myeloid cells of various maturation stages in peripheral blood and bone marrow, is caused by the oncoprotein BCR-ABL1, a dysregulated tyrosine kinase^1^. In early 2001, the first targeted therapy against an oncoprotein, the magic bullet imatinib mesylate, was introduced^2^. In the following years, multiple different second- and third-generation tyrosine kinase inhibitors (TKIs), as well as asciminib, an allosteric inhibitor binding to the myristoyl site^3^, which is now in clinical trials, emerged as even more potent drugs against the BCR-ABL1 oncoprotein. These drugs are successful at inducing hematologic, cytogenetic, and molecular remissions in the majority of patients, leading to a normal life expectancy. However, CML stem cells, which are independent of the activity of BCR-ABL1^4,5^, express the markers CD26^6^, interleukin-1 receptor accessory protein (IL1RAP)^7^, and CD93^8^, and are the cause of disease relapse and progression to blast crisis (BC) (Table 1), are not eradicated by TKIs^5,9,10^. In addition, CML cells employ other mechanisms to evade TKIs, for instance by way of molecular resistance^11^. Given the high costs of maintenance therapy with TKIs, as well as associated side effects, the goal has now become to discontinue TKIs in patients who have achieved and maintained a ≥4 log reduction in *BCR-ABL1* transcripts, representing molecular response (MR) 4, for at least 2 years.

**Table 1.** Characteristics of the three different phases of chronic myeloid leukemia (CML): chronic phase (CP), accelerated phase (AP), and blast crisis (BC).

Alterations	CP	AP	BC
**Oncogene**	*BCR-ABL1*	*BCR-ABL1*	*BCR-ABL1*
**Blast count**	<10%	10–19% in the peripheral blood and/or bone marrow	>20%
**Cell of origin**	Hematopoietic stem cell		Hematopoietic stem or progenitor cell
**Additional chromosomal** **alterations**		Second Ph, trisomy 8, isochromosome 17q, trisomy 19, complex karyotype, or abnormalities of 3q26.2^28^	Trisomy 8, isochromosome 17, duplication of the Ph chromosome or chromosome 19, 21, or 17, loss of chromosome Y or monosomy 7^29^
**Epigenetic factors**			*ASXL1*, *DNMT3A*, *RUNX1*, and *TET2*^30^
**Tumor suppressors**			*RB1*, *TP53*, and *CDKN2A*^29^
**Other kinase involvement**			Fyn kinase, CaMKIIγ^31,32^
**DNA damage response**	Relatively low		Impaired^33^

*Abbreviations*: ASXL1, ASXL transcriptional regulator 1; CaMKIIγ, calcium/calmodulin dependent protein kinase II gamma; CDKN2A, cyclin dependent kinase inhibitor 2A; DNMT3A, DNA methyltransferase 3 alpha; Ph, Philadelphia chromosome; RB1, RB transcriptional corepressor 1; RUNX1, RUNX family transcription factor 1; TET2, Tet methylcytosine dioxygenase 2; TP53, tumor protein P53.

Therefore, in view of the non-eradication of CML stem cells by TKIs, whose necessity is controversial^12^, and the aim of achieving treatment-free remission (TFR) after discontinuation of TKIs, multiple studies have focused on novel pathways influencing CML leukemic stem cell (LSC) maintenance and CML progression to BC. Although our work is far from complete, our understanding of factors maintaining TFR, such as the immune system or the bone marrow microenvironment (BMM), has grown considerably, and various strategies for combinatorial therapies to target and eradicate CML stem cells are being tested.

## Treatment-free remission

TKIs have certain off-target toxicities, such as vascular events, pleural effusions, etc.^13–16^. Certain drug interactions have been reported, and cessation of TKI therapy is advised in young women who wish to become pregnant. In addition, co-payments by patients and healthcare costs for lifelong therapy are significant^17^.

**Figure 1.**
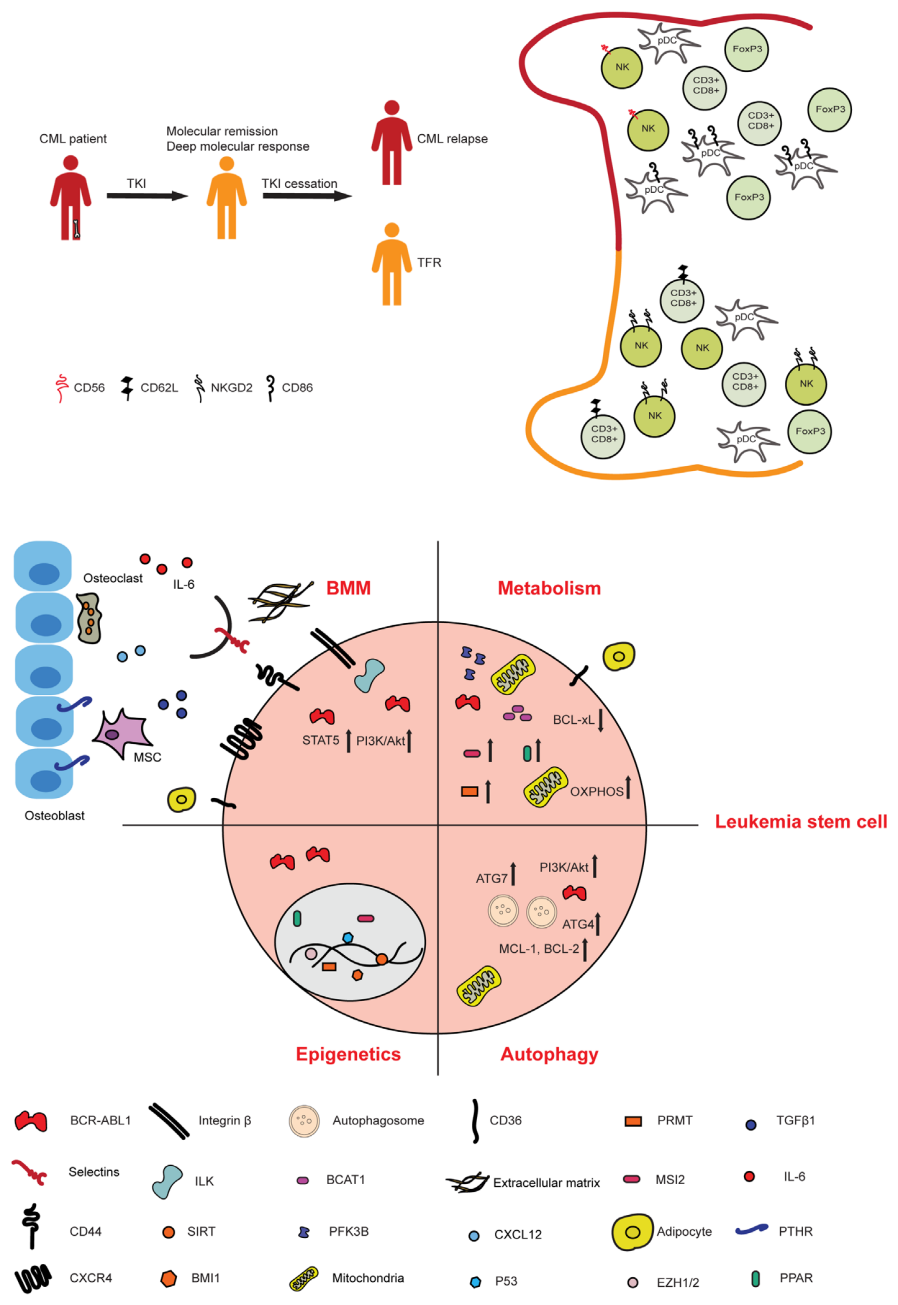
Factors influencing the persistence of CML LSC and TFR. A) Schematic representation of the possibilities of the course of a CML patient undergoing TKI treatment and, consequently, cessation of TKI treatment. Stopping TKIs may be followed by relapse or TFR. The various immune cell types and their respective receptors contributing to relapse versus TFR are depicted. B) Molecular and cellular components influencing the persistence of leukemic stem cells relating to the BMM, metabolism, epigenetics, and autophagy are shown. Factors in or on CML stem cells like cellular receptors, signaling components, and pathways influencing TFR are also represented. The BMM in CML consists of various cell types, secreted factors, and components of the extracellular matrix. *Abbreviations*: ATG, autophagy-related protein; BCAT, branched-chain amino acid aminotransferase; Bcl-xL, B-cell lymphoma-extra large; BMI1, B lymphoma Mo-MLV insertion region 1 homolog; BMM, bone marrow microenvironment; CD62L, L-selectin; CML, chronic myeloid leukemia; CXCL12, C-X-C motif chemokine ligand 12; CXCR4, C-X-C chemokine receptor type 4; EZH, enhancer of Zeste homolog; FOXP3, forkhead box P3; IL, interleukin; ILK, integrin-linked kinase; MSC, mesenchymal stromal cell; MSI2, Musashi-2; NK, natural killer; OXPHOS, oxidative phosphorylation; pDC, plasmacytoid dendritic cell; PFK3B, 6-phosphofructo-2-kinase/fructose-2,6-biphosphatase 3; PI3K, phosphoinositide 3-kinase; PPAR, peroxisome proliferator-activated receptor; PRMT, protein arginine N-methyltransferase; PTHR, parathyroid hormone receptor; SIRT, sirtuins; STAT5, signal transducer and activator of transcription 5; TFR, treatment-free remission; TGFβ1, transforming growth factor β1; TKI, tyrosine kinase inhibitor.

In 2007, a French CML study showed that 50% of patients who had been negative for *BCR-ABL1* transcript for approximately 2.5 years, in whom TKI therapy was discontinued, did not relapse 18 months after TKI cessation^18^. This prompted discussion about TFR, which was followed up by further clinical trials with, overall, similar results^19–26^ (Figure 1A).

## Control of TFR by immunological mechanisms

Concentrated efforts are now directed at understanding the cellular and molecular factors influencing TFR. Increasing evidence, including the finding that the immunomodulatory agent interferon *α* may improve the maintenance of TFR^27^, is suggestive of immunological contributions to obtaining and maintaining TFR. In the EURO-SKI trial, an increased proportion of NK cells was found to be associated with longer relapse-free survival. Patients with a more immature NK cell phenotype (CD56^bright^) were less likely to maintain TFR, and secretion of tumor necrosis factor *α* and interferon *γ* correlated with the success of discontinuation of TKIs^34^. Another study found an increased number of NK cells and a reduction of the CD3^+^ CD8^+^ CD62L^+^ cell fraction in patients in whom imatinib was stopped compared to patients still receiving imatinib^35^. Patients maintaining TFR had a higher proportion of NK cells expressing activating NK cell receptors, such as NKG2D, but decreased expression of the inhibitory NK cell receptors KIR2DL2/DL3/DS2^36^ and a lower percentage of FoxP3^+^ regulatory T cells and monocytic myeloid-derived suppressor cells, suggesting that these factors may be used as a predictive scoring system^37^. In another prospective analysis expression of CD86 (B7.2), the ligand for T-cell inhibitory receptor (CTLA-4), on plasmacytoid dendritic cells (pDCs) was found to be predictive of TFR. An increased number (>95) of CD86^+^ pDCs per 10^5^ lymphocytes was associated with reduced relapse-free survival (30%), while reduced numbers (<95) of CD86^+ ^pDCs per 10^5^ lymphocytes were associated with increased (70%) TFR^38^ (Figure 1A). A more thorough understanding of the cellular and possibly inflammatory contributions of the immune system and its modulations by medications may increase TFR rates in future.

## Novel and potentially targetable mechanisms contributing to the maintenance of CML LSCs

### Epigenetics

With the emergence of sophisticated technologies like (single cell) DNA or RNA sequencing, epigenomics, metabolic profiling, lipidomics, etc., we are beginning to understand and identify novel proteins and pathways that CML cells and CML LSCs employ during disease progression, TKI resistance, and relapse. Some epigenetic modifications, meaning heritable changes in the function of genes without alteration of the gene sequence, such as aberrant DNA methylation, play an important role during CML initiation and development^39^. Furthermore, the polycomb group of proteins, consisting of polycomb repressive complex 1 and 2 (PRC1/2), have been identified as crucial modulators of CML pathogenesis by mediating the methylation of histone H3, initiated by the catalytic subunit of PRC2, EZH2. The deregulation of PRC2 in CML LSCs led to epigenetic modification at H3K27, while inhibition of EZH2 or treatment with TKIs significantly increased methylation at H3K27. Combination treatment with an EZH2 inhibitor and TKIs potentiated H3K27 methylation, leading to reduction of LSCs and improving outcome in xenotransplantation studies compared to treatment with TKIs alone^40^. Another study identified EZH2 as being upregulated in CML LSCs, where it is essential for colony formation, cell survival, and cell cycling. Conditional loss of *Ezh2* impaired CML initiation and maintenance^41^. B lymphoma Mo-MLV insertion region 1 homolog (BMI1) is a transcriptional repressor of components of PRC proteins. Expression of BMI1 RNA was found to be higher in advanced phases of CML and was considered a possible predictive marker for overall survival of CML patients^42^. Similarly, retroviral transduction of human CD34^+^ CML cells with BMI1 and transplantation into immunosuppressed mice revealed a collaborative effect of BMI1 and BCR-ABL1, leading to increased self-renewal and proliferation capacity^43^.

Protein arginine methyltransferase 5 (PRMT5) catalyzes the transfer of methyl groups to arginine and is essential for normal HSC maintenance and hematopoiesis^44^. PRMT5 was highly expressed in CD34^+^ cells from CML patients in a STAT5-dependent manner, and shRNA-mediated silencing of *Prmt5* or its inhibition in a murine model of CML significantly prolonged survival and impaired the self-renewal capacity of LSCs^45^.

Histone deacetylases belong to another class of epigenetic proteins causing the deacetylation of histones, thereby regulating gene expression. Sirtuins (SIRT1) are NAD-dependent deacetylases upregulated in CML cells. They promote CML cell survival and proliferation by deacetylating SIRT1 substrates like FOXO, p53, and Ku70. Deficiency of SIRT1 or treatment with the SIRT1 inhibitor tenovin-6 suppressed CML induction in a murine model. Imatinib treatment led to partial reduction of SIRT1 expression, and inhibition of SIRT1 sensitized CML cells to imatinib^46^. Similarly, inhibition or knockdown of SIRT1 increased the apoptosis of CML LSCs and led to their growth reduction. This effect was mediated by the acetylation and transcription of p53 in CML progenitor cells^47^ (Figure 1B). Combination of histone deacetylase inhibitors (HDACi) with TKIs effectively targeted quiescent LSCs and significantly reduced LSCs in a murine model^48^. Ineffectiveness or toxicity of some of these therapies in humans have limited their use, problems that may be rectified by second-generation agents.

### Autophagy

Autophagy is a central catabolic pathway employed by various malignancies^49^. BCR-ABL1-expressing cells have low levels of autophagy, while inhibition of the autophagy pathway drives apoptosis^50^. Autophagy is induced in CML cells as a means of cytoprotection upon treatment with TKIs^51^, and shRNA-mediated knockdown or pharmacological inhibition of autophagy in combination with TKIs led to the elimination of LSCs^52^. Another study demonstrated the synergistic effect of imatinib and the autophagy inhibitor spautin-1 in inducing apoptosis in CML cell lines. This was mediated by suppression of the PI3K/AKT pathway and downregulation of the anti-apoptotic proteins MCL-1 and BCL-2 via GSK3*β*^53^. Expression of the autophagic protein ATG4B, a component of the LC3 autophagosome system, was higher in CD34^+^ cells from CML patients, and expression differed between patients prior to therapy with TKIs as well as responders and non-responders to imatinib treatment^54^. Furthermore, knockdown of *ATG7* sensitized CML stem and progenitor cells to imatinib treatment via metabolic alterations in CML cells, increased production of reactive oxygen species, and increased differentiation of cells towards the erythroid lineage^55^ (Figure 1B). Indeed, the autophagy inhibitor hydroxychloroquine (HCQ) in combination with imatinib had moderate efficacy in reducing *BCR-ABL1* transcript levels in a clinical trial^56^. However, administration of a second-generation autophagy inhibitor, Lys05, in a murine model had superior effects on inhibiting autophagy in LSCs and reducing their quiescence and number in** comparison to HCQ. Lys05 was also shown to potentiate the effect of TKIs on patient-derived LSCs^57^. The quest for more potent, non-toxic autophagy inhibitors is ongoing.

### Metabolism

Cancer cells are characterized by altered metabolic demands required for rapid division and buildup of biomass. In the past decade, with novel and improved technologies, we have begun to understand the unique and important contribution of metabolism towards cancer development and progression^58^.

Examination of the metabolic status of primitive, stem cell-enriched CD34^+^ or CD34^+^ CD38^–^ cells compared to differentiated CD34^–^ cells from CML patients showed an increased oxidative status in the CML stem cell fraction. A combination of TKIs and tigecycline, an inhibitor of mitochondrial protein translation, had a superior effect on eradicating LSCs^59^. Using a novel approach combining high-sensitivity mutation detection with transcriptome analysis of single cells, researchers examined the stem cell population of CML patients throughout the disease course. A unique enrichment of components of metabolic pathways was found in BCR-ABL1^+^ compared to BCR-ABL1^–^ stem cells^60^. A combination of bioinformatic and RT-PCR analyses revealed the glycolytic enzyme 6-phosphofructo-2-kinase/fructose-2,6-biphosphatase 3 (PFKFB3) to be associated with TKI resistance in a kinase-independent manner. Knockdown or inhibition of PFKFB3 reduced CML cell growth, prolonged survival in a xenotransplantation model, and increased sensitization to TKIs^61^. Gonadal adipose tissue (GAT) was identified as a reservoir for LSCs, especially for a population of CD36^+^ LSCs of a pro-inflammatory phenotype in a murine model of BC CML. These LSCs induced lipolysis, which in turn induced fatty acid oxidation in LSCs, leading to evasion of chemotherapy^62^. The pyruvate kinase isozyme M2 (PKM2) was more highly expressed in TKI-resistant primary cells and CML cell lines. Inhibition of PKM2 reduced glycolysis, downregulated mTOR and the PI3K/AKT pathway, and in combination with TKIs induced apoptosis more efficiently than TKIs alone^63^. Furthermore, deficiency of PKM2 led to prolongation of survival in a murine model of CML^64^.

The nuclear peroxisome proliferator-activated receptors (PPARs) belong to a family of transcription factors regulating cellular proliferation, differentiation, and metabolism^65^. The PPARγ agonist pioglitazone effectively targeted residual CML LSCs via a decrease of STAT5 expression, as well as its targets HIF2*α* and CITED2. This mechanism was independent of BCR-ABL1 activity. Treatment of three patients with pioglitazone, in addition to imatinib, led to a complete molecular response of up to almost 5 years, even after the removal of pioglitazone^66^.

With regard to protein metabolism, CML LSCs are enriched for certain dipeptides, which activate amino acid signaling and thereby maintain stemness via p38MAPK–Smad3 signaling^67^. The enzyme branched-chain amino acid transferase (BCAT1) was upregulated in CML cells and promoted the formation of branched chain amino acid (BCAA) production in CML cells during disease progression. Musashi2 (MSI2), an RNA-binding protein, acted upstream of BCAT1, regulating its expression (Figure 1B). Blocking BCAT1 expression or activity led to the differentiation of CML cells and impaired progression of BC CML^68^. Future experiments need to accurately model the metabolic complexities within the tumor microenvironment, such as hypoxia and nutrient deprivation, and address the impact of leukemia-induced metabolic alterations on normal HSCs.

### Bone marrow microenvironment

Leukemia (stem) cells, similar to their normal hematopoietic counterparts, depend on their microenvironment. The BMM, which is believed to provide shelter from chemotherapy and TKIs via its specific cellular and molecular architecture, influences TFR. Reciprocally, the BMM is modulated by leukemia cells. Differences in adhesion between CML LSCs and normal HSCs with regard to the β integrins, which may be modified by treatment with IFN*α*^69^, or CD44^70^ or their binding to E-selectin^71^ amongst other ligands, have long been appreciated^72^. Expression of integrin β3 was found to be increased in CML cells resistant to imatinib due to the *BCR-ABL1^T315I^* point mutation. Knockdown or pharmacological inhibition of integrin-linked kinase (ILK), a protein of the adhesosome complex downstream of the integrins and upregulated in *BCR-ABL1^T315I^*^+^ cells, significantly delayed CML progression *in vivo* via increased levels of fibronectin, an extracellular matrix protein, in the BMM. Prolongation of survival in this CML model could also be achieved by the administration of fibronectin^73^. Expression of ILK was higher in LSCs of TKI non-responder patients, and inhibition of ILK sensitized these cells to TKIs. Furthermore, ILK was required for maintaining an essential level of oxidative phosphorylation in CML LSCs^74^. Kindlins are other components of the focal adhesosome and molecular interactors of integrins. Loss of kindlin 3 (K3) increased the release of CML LSCs from the bone marrow and inhibited the maintenance and survival of leukemia cells^75^.

Inhibition of the interaction of CD44 with E-selectin by GMI-1271, an E-selectin inhibitor, in combination with imatinib led to greater elimination of LSCs in a murine model of CML. Non-adhesion to E-selectin led to an increased cell cycle in CML LSCs with a concomitant upregulation of the transcription factor SCL/TAL-1, which further negatively regulated CD44 expression and potentiated the effect of the combinatorial treatment^76^.

The C-X-C chemokine receptor type 4 (CXCR4) is downregulated on CML cells, while its expression is upregulated in the presence of imatinib, increasing migration towards stroma and inducing G0-G1 cell cycle arrest^77^. Inhibition of CXCR4 using plerixafor inhibited cell migration and adhesion of CML cells to stroma. *In vivo,* plerixafor mobilized leukemia cells and in combination with nilotinib significantly reduced tumor burden^78^. The expression of C-X-C motif chemokine ligand 12 (or stromal derived factor 1*α*), the ligand for CXCR4, is reduced in the CML BMM due to increased secretion of granulocyte colony-stimulating factor adding to reduced retention of CML LSCs in the BMM^79^. Additionally, deletion of CXCL12 from mesenchymal stromal cells (MSCs) in the BMM led to upregulation of EZH2 function in LSCs in the form of enhanced self-renewal and expansion^80^.

Expression of the pro-inflammatory cytokine IL-6 is regulated by BCR-ABL1. Expression of IL-6 activates a paracrine loop, inducing myeloid lineage commitment in CML multipotent progenitors and sustaining CML pathogenesis^81^. In a process mediated by IL-6 after exposure to CML cells, normal hematopoietic progenitor cells acquired the genetic signatures of the malignant cells, altering the differentiation, reducing the ability for self-renewal, and increasing the division of normal cells. Treatment with an anti-IL-6-blocking antibody inhibited these effects and significantly reduced tumor burden^82^.

Furthermore, activation of the parathyroid hormone (PTH) receptor on osteoblastic cells suppressed CML induction in a murine model. This effect was mediated by increased levels of transforming growth factor (TGF) β1 released from the BMM. Consistently, knockdown of the receptor for TGF β1 (*Tgfbr1*) on CML cells suppressed the effect of PTH receptor activation on osteoblastic cells^83^ (Figure 1B). Efforts must be undertaken to culture and analyze niche cells from patients with hematological malignancies and/or to create artificial niches for in-depth study of these intricacies.

## Novel mechanisms involved in driving progression to BC

Additional genetic alterations stimulated by the aberrant activity of BCR-ABL1 on the generation of reactive oxygen species and the impairment of DNA repair mechanisms advance chronic phase disease to BC^33^. The cell of origin in BP CML, similar to acute myeloid leukemia, is thought to be a hematopoietic stem or progenitor cell^9^. Most common chromosomal abnormalities reported in BC involve chromosomes 17, 8, or 19 or the Philadelphia (Ph) chromosome, which harbors the BCR-ABL1 fusion itself, while, molecularly, the MDS1 and EVI1 complex locus (*MECOM*) gene and tumor suppressors like *RB1*, *TP53*, and *CDKN2A* are frequently affected^29^. Additional genetic lesions have also been reported in Ph^–^ and Ph^+^ cells in BC, with commonly affected genes including *ASXL1*, *DNMT3A*, *RUNX1*, and *TET2*, although these may have been due to clonal hematopoiesis^30^. Members of the Src kinase family were also upregulated during the BC phase of CML. In particular, increased Fyn kinase levels were associated with increased aggressivity and genomic instability in CML and targeting Fyn sensitized cells to TKIs^31,32^. Calcium-calmodulin-dependent kinase IIγ (CaMKIIγ) expression was also positively correlated with BC CML by regulating the activity of p27Kip1 (Table 1). Downregulation of CaMKIIγ inhibited disease progression while overexpression aggravated disease development in a murine model of BC CML^84^. An ATP-competitive inhibitor of CaMKIIγ, berbamine, induced apoptosis in TKI-resistant K562 cells^85^. However, further mechanisms driving the progression of CML to BC are largely obscure, partly because of the absence of cell lines and physiological models of BC, and remain a major focus of current efforts.

## Conclusion

Taken together, while many believe CML to be curable, this does not seem to be the case, at least in the majority of patients, and several conundra persist. Current and future efforts in CML research are focused on the novel concept of TFR, as well as on LSC-intrinsic factors like metabolism, epigenetics, or autophagy or LSC-extrinsic factors such as the immune system or the BMM influencing TFR. Additionally, we are faced with BC, which remains a frequently fatal condition, despite the myriad of drugs now at our disposal. While great strides have been made in understanding and treating CML, which has acted as a model disease for many types of cancers, many questions remain. These will be tackled in future, paving the way also for the understanding of other cancers, as has become custom for CML.
